# Epigenetic Regulation of Viral Biological Processes

**DOI:** 10.3390/v9110346

**Published:** 2017-11-17

**Authors:** Lata Balakrishnan, Barry Milavetz

**Affiliations:** 1 Department of Biology, Indiana University Purdue University Indianapolis, Indianapolis, IN 46202, USA; latabala@iupui.edu; 2 Department of Biomedical Sciences, University of North Dakota School of Medicine and Health Sciences, Grand Forks, ND 58203, USA

**Keywords:** epigenetic, DNA virus, regulation, gene expression, histone modifications, nucleosomes, DNA methylation, ChIP-Seq, latency

## Abstract

It is increasingly clear that DNA viruses exploit cellular epigenetic processes to control their life cycles during infection. This review will address epigenetic regulation in members of the polyomaviruses, adenoviruses, human papillomaviruses, hepatitis B, and herpes viruses. For each type of virus, what is known about the roles of DNA methylation, histone modifications, nucleosome positioning, and regulatory RNA in epigenetic regulation of the virus infection will be discussed. The mechanisms used by certain viruses to dysregulate the host cell through manipulation of epigenetic processes and the role of cellular cofactors such as BRD4 that are known to be involved in epigenetic regulation of host cell pathways will also be covered. Specifically, this review will focus on the role of epigenetic regulation in maintaining viral episomes through the generation of chromatin, temporally controlling transcription from viral genes during the course of an infection, regulating latency and the switch to a lytic infection, and global dysregulation of cellular function.

## 1. Introduction

The words of the Nobel Laureate Bob Dylan “Because something is happening here, but ya don’t know what it is, do you Mister Jones?” could very well apply to our understanding of the role that epigenetics plays in regulating viral life cycles. While there is lots of evidence that epigenetics plays a critical role in the regulation of virus infections, in many cases the details of the role have not been completely elucidated. While there are likely to be many factors contributing to our relative lack of understanding of the details of viral epigenetic regulation, one of the major contributors is the inherent complexity of epigenetic regulation. There are presently five well-characterized processes that are thought to make up epigenetic regulation including: DNA methylation, nucleosome positioning, histone variants, histone modifications, and regulatory RNA (Table 1). Needless to say, epigenetic regulation has been the subject of intense interest because of its potential ability to maintain stable gene expression when necessary with the flexibility to respond to changes in environment. Epigenetic regulation contributes to stability by maintaining the chromatin structure of the genome from parental to daughter chromatin during replication and by extension the gene expression patterns by what is known as transgenerational epigenetic regulation. At the same time, each of the five epigenetic processes are sufficiently plastic to allow for the establishment of new epigenetic states as a result of changes in the environment such as the de novo expression of new transcription factors by generating new chromatin structures. Because of the relatively limited coding capacity of a typical DNA virus genome, the viruses tend to use one or more cellular biological processes to accomplish their own molecular biology. As a result, epigenetic regulation during viral infections is usually bidirectional between the virus and the cell. The virus will use cellular factors for transcription or replication and epigenetic cofactors such as histone acetylases, deacetylases, methyases, and demethylases in the control of its life cycle, The virus may also epigenetically dysregulate cellular pathways in order to optimize its own transcription or replication or evade the cells innate immune response.

### 1.1. New Technology to Study Viral Epigenetics

Two relatively recent technological advances have substantially contributed to our understanding of epigenetic regulation. The first, chromatin immunoprecipitation (ChIP) [1,2,3] made it possible to determine which histone variants, histone modifications, or other proteins were bound to chromatin isolated under specific conditions of gene expression. Combining ChIP analyses with the more recently developed next-generation sequencing in a ChIP-Seq [4] made it possible to determine the genomic location of nucleosomes in general and more specifically nucleosomes which contain a particular histone variant or histone modification over a complete genome. ChIP-Seq also can be used to determine the genomic location of proteins of interest such as the steady state binding sites for RNA Polymerase II (RNAPII). Together, these techniques used with various model systems have led to certain general rules with respect to the function of DNA methylation, nucleosome location, and histone modifications.

### 1.2. Role of Epigenetic Regulation

DNA methylation is generally thought to be associated with repression of transcription [5]. Nucleosome location is thought to play a critical role in regulation by controlling the availability of DNA sequences targeted by transcription factors or RNAPII [6]. Typically, a binding site will be covered by a nucleosome during repression and uncovered during activation. The rules for histone modifications are somewhat more complex due to the inherent complexity of histone modification. In general, histone H3 and H4 acetylation are associated with active chromatin as is histone H3K4 methylation and H3K36 methylation [7,8]. In contrast, histone H3K9 methylation and H3K27 are associated with repression [9,10,11]. Histone H4K20 methylation is somewhat more complex, having been reported associated with both activation and repression (reviewed in [12]). With the advent of ChIP-Seq, it is clear that these rules are actually more complex, because the position of nucleosomes in a genome that carry either mono-, di-, or tri-methylated histone may be different, suggesting that the pattern of methylated nucleosomes may have different functions.

### 1.3. Why Do Viruses Use Epigenetic Regulation?

DNA viruses, which exist as nuclear episomes (polyomaviruses, adenoviruses, papillomaviruses, herpesviruses, and hepatitis B virus), are very likely to exploit epigenetic mechanisms to regulate biological activities during their life cycle for a number of reasons. First, these viruses typically exist as chromatin either throughout their life cycle or at least when present in the nucleus of a cell. Second, the viruses usually utilize a number of cellular regulatory factors, co-factors, and enzymes to accomplish gene expression and its regulation. Finally and importantly, these viruses require epigenetic or similar processes to allow the different viral genome states necessary to complete an infection to coexist. Many DNA viruses are known to coordinately perform divergent biological processes within the same infected nucleus of a cell. For example, at later times in a typical infection by a DNA virus one might expect to find viral genomes replicating, transcribing one or more distinct genetic units, and also undergoing encapsidation to form new virus particles. Each one of these distinct biological processes would be expected to be tightly controlled relative to the other processes and yet would all be based upon the same DNA sequence of the virus. Differences in one or more epigenetic process listed above would allow for the coexistence of the different functional forms of the viral genome and maintain the necessary tight control. The viruses may also epigenetically dysregulate host cell biology in order to enhance their own biological processes. For example, they may stimulate the synthesis of factors involved in DNA replication or transcription or alternatively inhibit pathways of immune surveillance.

This review will focus on DNA viruses which exist as episomes for at least part of their life cycle and will update a prior publication [13] with data which has been published since January 2015. It will focus primarily on members of the polyomavirus, adenoviruses, papillomaviruses, herpesvirus, and hepatitis B virus all of which have been relatively extensively studied to date. Since many of these viruses also appear to affect their target cells by disruption of cellular epigenetic regulatory pathways, this aspect of epigenetics will also be addressed.

## 2. Polyomaviruses—Simian Virus 40

The polyomaviruses are a family of small (<10 kb) double-strand closed-circular DNA tumor viruses [14,15]. Based upon work with Simian Virus 40 (SV40), a well-studied member of this family, it has been established that the viruses exist as typical eukaryotic chromatin when found in the nucleus of infected cells and in the virus particle. Because the genome of these viruses is organized into chromatin, it seemed likely that the viruses would undergo the typical forms of epigenetic regulation. Using SV40 as a model for the family, it has been shown that the viruses undergo almost all of the same forms of epigenetic regulation as cellular chromatin, including nucleosome location [16], histone modifications [17,18], and miRNAs [18]. However, polyomaviruses do not appear to utilize DNA methylation as a form of epigenetic regulation due to their small size and relative absence of target sites for methylation. However, it is possible that an infection by a polyomavirus could result in methylation of cellular DNA. To date, there have been no studies addressing epigenetic changes to the cell following infection by SV40 or mouse polyomavirus. Like SV40, mouse polyomavirus has also been shown to undergo histone modification [19]. Because the SV40 genome exists as chromatin throughout its life cycle epigenetic regulation is likely involved in all aspects of its molecular biology. This likely includes regulatory information in the chromatin of the virus to direct the establishment of a new infection as well as regulating the relative levels of early and late transcription, the proportion of minichromosomes entering replication, and ultimately the removal of minichromosomes by encapsidation.

Interestingly, epigenetic regulation of SV40 was first identified in the late 1970s, although the changes in nucleosome positioning around the SV40 promoter [20,21,22,23,24,25] and histone modification by acetylation [26,27] were not recognized as epigenetic regulation at the time. More recently, SV40 has been used as a model system to study basic aspects of epigenetic regulation and the role of epigenetics in regulating the SV40 life cycle focusing primarily on changes in histone modifications [17,18,28,29,30,31,32,33,34,35,36]. While informative, these studies did not yield a complete picture of regulation because they were based primarily on the association between biological events and the changes in histone modifications. They were also limited because they did not address the important relationship between nucleosome positioning and histone variants or modifications.

In order to address these two issues we have taken a three-stage approach. In the first stage, we have determined the global location of nucleosomes in SV40 chromatin isolated at different times in infection and from virus particles using micrococcal nuclease to prepare sequencing libraries and next generation sequencing (NGS). This recently published work [37] will be discussed in more detail below. In the second stage we are using ChIP-Seq with SV40 chromatin isolated at 48 h post-infection and from virus particles to determine the global location of nucleosomes which contain specific histone tail modifications including hyperacetylated H3, hyperacetylated H4, H3K4me1, H3K4me2, H3K4me3, H3K9me1, H3K9me2, H3K9me3, and H4K20me1. We have previously shown that all of these histone tail modifications are present in SV40 chromatin. Since these studies are in progress, we will only briefly describe them. While these first two stages have yielded or are yielding very interesting results concerning nucleosome positioning and their related histone modifications, the studies have utilized mixed populations of SV40 chromatin naturally present at the time that the chromatin was isolated. Because this chromatin is inherently heterogeneous, it is still difficult to ascribe specific elements of chromatin structure to specific biological activities. In the third stage, we will use a strategy that we previously developed to determine the location of nucleosomes containing specific histone tail modifications in specific forms of biologically active SV40 chromatin including transcribing, replicating, and encapsidating chromatin. We will briefly describe this strategy and its utility at the end of this section.

**Figure 1:**
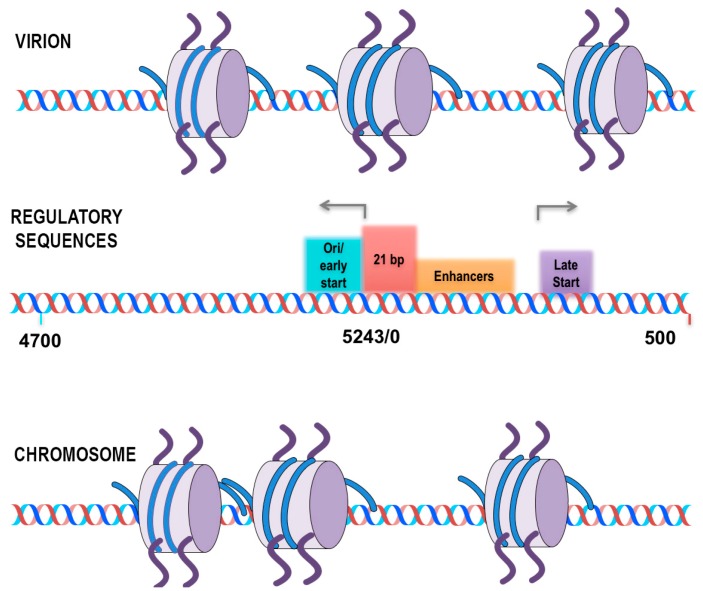
Schematic representation of the position of nucleosome in the major forms of SV40 chromatin present in virion and minichromosomes isolated at 48 h. Location of nucleosomes in SV40 chromatin from disrupted virion (Top) and minichromosomes isolated at 48 h post infection (Bottom) are shown. The regulatory sequences provide visual alignment for the position of the identified location of nucleosomes.

SV40 chromatin was analyzed for the location of all nucleosomes on the SV40 genome using micrococcal nuclease to generate nucleosome-sized fragments of DNA. Because of the large number of reads that can be obtained from SV40 chromatin, the data was analyzed without having to bin the reads, which allowed for mapping to the genome with essentially single base resolution. Based upon this analysis of global nucleosome positioning, a model was proposed in which nucleosomes play a critical role in regulating transcription during the SV40 life cycle. In chromatin from virus particles, nucleosomes were observed to be present at nt 5223 and nt 363 effectively covering the major early and late start sites for transcription. In contrast, in chromatin from minichromosomes isolated at 30 min and 48 h post-infection, nucleosomes were found at nt 5119 and nt 212, exposing the early and late start sites. The inhibition of transcription by the nucleosomes blocking the early and late transcription start sites was further confirmed by analyzing the SV40 mutant cs1085. This mutant lacks T-antigen binding Site I and as a consequence does not downregulate early transcription. If the nucleosome located at nt 5223 was involved in blocking early transcription one would expect to see that, in the mutant, the nucleosome would be absent. Interestingly, both the nucleosome at nt 5223 and the one located at nt 363 were substantially reduced on the SV40 chromatin in the mutant. While this was consistent with the expectation for the early transcription it was unexpected that the late start site would also be affected [37]. This regulatory model is shown in Figure 1.

In addition to the changes in the promoter/regulatory region of SV40, changes in the nucleosome location around the 5′ end of the early splice site, nt 4885, and near the termination of transcription, nt 2800–2900 were also observed in this study. The significance of these changes were not further explored in the publication [37]. Nucleosome positioning near the termination of transcription and in translation control regions has previously been observed in cellular chromatin (reviewed in [38]) but not in viral chromatin.

In the second stage of our studies we are mapping onto the SV40 genome the location of nucleosomes present in SV40 minichromosomes and virions, which contain each of the histone modifications described above. From this analysis, we expect to answer a number of questions related to nucleosome organization and regulation. First, we will determine whether there are preferred locations on the genome for nucleosomes containing each of the specific tail modifications. If, as expected, preferred locations are found for one or more of the histone modifications, we will then characterize the relationships between each preferred location, the underlying DNA sequences, and the location of all nucleosomes as determined in our recent publication [37]. This will allow us to determine whether the nucleosomes which we have identified recently which appear to regulate early and late transcription by their presence or absence carry specific histone modifications, which could contribute to how their presence is regulated. This analysis will also allow us to determine whether the preferred location of a particular histone modification corresponds to the preferred location of any other modification or whether preferred locations are associated with distinct nucleosome locations.

**Figure 2:**
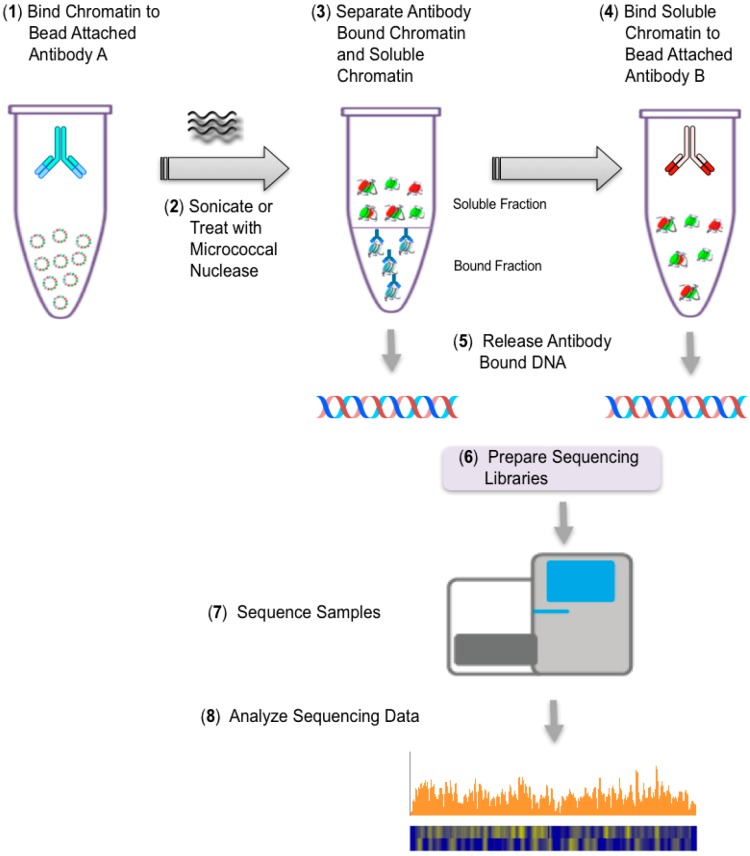
Schematic of protocol for immune selection fragmentation followed by immunoprecipitation (ISFIP) and next-generation sequencing (NGS).

The third stage of these studies is based upon the strategy shown in Figure 2, which, we have previously used successfully to identify histone modifications in SV40 chromatin which is bound by RNA Polymerase II (RNAPII) [31,32,33] or replication-associated proteins [34]. In these future studies, we will determine the location of nucleosomes containing modified histones in transcribing minichromosomes. Transcribing SV40 minichromosomes are being immune-selected with antibody to RNAPII as in a typical ChIP. Following sonication of the bound chromatin, the agarose containing the chromatin fragments bound with RNAPII antibody are separated from the fragments lacking RNAPII. The latter are then subjected to a second ChIP with an antibody to a histone modification of interest. In our initial studies we are using antibody to hyperacetylated H3 and hyperacetylated H4 since we have previously shown that chromatin fragments containing these modified histones are present in SV40 minichromosomes also containing RNAPII [31,32].

## 3. Other Polyomaviruses

None of the other polyomaviruses—such as John Cunningham (JC) virus, BK virus, or Merkel cell virus—have been studied to the same extent as SV40 with respect to epigenetic regulation. Since the viruses are similar to SV40 they are likely to use epigenetics in the same way to regulate infection and the various functional forms of the viral minichromosome. There has been recent progress in investigating the epigenetics of these viruses with respect to miRNAs, histone modification, and the epigenetic reader, BRD4, primarily in explaining how these viruses may dysregulate infected cells. Many if not all polyomaviruses contain a miRNA in the late message strand which can be cleaved to yield a miRNA which inhibits expression of the early genes (reviewed in [39]). This miRNA shares sequence homology with certain cellular miRNAs and it has been suggested that the miRNA could dysregulate cellular genes as well [39]. Two recent publications support this suggestion [40,41]. The former presents evidence for the transfer of JC virus miRNA in exosomes from infected cells to uninfected cells while the latter demonstrates a link between miRNA production by raccoon polyomavirus and the development of tumors in animals.

A role for global dysregulation of histone modification in cancers following transformation by Merkel cell polyomavirus has also been recently described [42]. A strong correlation was found between tumors that expressed the large T-antigen of Merkel Cell virus and repression of H3K27 trimethylation (H3K27me3). Because repression of H3K27me3 would be associated with increased transcription of normally silenced genes, the results suggested that Merkel cell virus could have its tumorigenic effect through this global dysregulatory pathway in susceptible cells.

BRD4 is thought to serve as a scaffold for activating transcription by binding to acetylated lysines present on histones in chromatin regions available for transcription and also binding to transcription factors necessary to initiate transcription. The function of BRD4 in the reactivation of JC virus in glial cells was investigated using either a BRD4 inhibitor or by mutating lysines targeted for acetylation by BRD4 on the transcription factor NF-κB p65 [43]. Since both the BRD4 inhibitor and the mutations blocked transcription of the early genes and reactivation of infection, epigenetic regulation by histone acetylation appears to play a critical role in the regulation of early JC virus transcription.

## 4. Adenovirus

The adenoviruses are a family of double-strand linear DNA viruses approximately 35 KB in length [44]. Within the virus particle adenovirus DNA is associated with a viral protein called protein VII which is a basic histone-like protein (reviewed in [45]). Protein VII appears to serve as a histone substitute to compact the adenovirus DNA in the virus particle. It is generally thought that following infection, the protein VII is replaced at least partially if not totally on the adenovirus genome by cellular histones. Because of its relatively large size and apparent absence of chromatin in the virus particle, it seems likely that the primary function of epigenetics is to regulate the various intracellular aspects of the virus life cycle. Although there are no ChIP-Seq based publications to date demonstrating this, there are several laboratories working on this subject.

The recent adenovirus publications focusing on epigenetics have addressed epigenetic effects on the host cell as a consequence of infection [44,45,46,47]. These studies can be divided into those that address global effects on cellular epigenetics and those that are specific to the effects of protein VII. The global effects on histone modifications were addressed using a mass spectrometry (MS) approach to identify post-translational histone modifications [48]. Infected cells were isolated at different times during infection and the histones prepared for MS analysis. The analysis demonstrated that there were distinct global changes in histone modifications during the course of an infection. However, the technique did not allow for the localization of the changes to specific genes.

The global effects on cellular transcription and histone modifications was also analyzed using the adenovirus early regulatory protein E1A wild-type and mutant viruses in the context of infection [47]. In this study, ChIP-Seq was used to identify the target genes, which underwent significant changes in transcription as measured by changes in histone acetylation. A number of genes, which would normally be expected to inhibit infection, were found to be repressed by E1A expression [47].

Our understanding of the epigenetic role of adenovirus protein VII was recently extended in a publication combining biochemistry, cell biology, and mass spectrometry [48]. Protein VII was shown to function in a manner similar to epigenetic readers by binding to high mobility group (HMG) proteins to prevent the loss of the HMG proteins from nucleosomes. Interestingly, the binding to nucleosomes was also dependent upon the post-translational modifications of protein VII again showing similarities to cellular epigenetic regulators [48].

## 5. Human Papillomaviruses

The human papillomaviruses (HPV) are a family of closed-circular double-stranded DNA viruses with an approximately 8000 bp genome [44]. Because of the similarity between HPV and the polyomaviruses with respect to their physical structure and genetics, HPV would also be expected to contain typical chromatin structure throughout its life cycle and to be regulated epigenetically. However, for technical reasons associated with its complex life cycle it has been much harder to study the role of epigenetics of the virus in a lytic infection and consequently there is much less known about the epigenetics of infection. The technical difficulties associated with studying the HPV life cycle result from the fact that the life cycle is regulated by the normal differentiation process of the infected epithelial cells. Since this is generally studied in relatively small raft cultures, it has been very difficult to obtain sufficient materials at different stages of the life cycle to study. There are examples of transformed cells derived from cancers in which the HPV exists as a nonintegrated episome, which can be studied, but in general it is not clear that epigenetic regulation in these examples necessarily mirror the normal processes which occur in a lytic infection.

Because HPV is a very important human pathogen causing a number of different types of cancer there is much more known about the role of epigenetics in the transformation of normal cells and in cancers. This role has been recently reviewed and will not be discussed in detail [49]. Not surprisingly, HPV appears to dysregulate a number of epigenetic processes during transformation and cancer including DNA methylation, histone modification, chromatin remodeling, and miRNAs [49]. Epigenetic dysregulation can occur either on cellular genes or on the viral genes, particularly when the virus is integrated into the host genome. Not unexpectedly, the primary transforming proteins—E6 and E7—play a major role in much of the known dysregulation.

Despite the difficulties associated with studying epigenetic regulation in HPV lytic infections there has been some notable recent work [50,51,52,53,54,55,56,57,58]. By characterizing the effects of dysregulation of the deactylase, Sirtuin 1, a role for histone acetylation during HPV replication was established [50]. A role for acetylation during the HPV life cycle coordinating with cell differentiation was observed following studies of TIP60, an acetyltransferase [51]. A role for BRD4, the chromatin reader—which binds acetylated histones in the activation of early HPV transcription—has also been established [52,53,54,55]. Together, these results suggest that histone acetylation is likely to occur during the HPV life cycle with functions similar to what is occurring in cellular epigenetic regulation. There is also evidence that histone methylation occurs during the HPV life cycle. The human interferon-inducible protein IFI16 appears to inhibit HPV replication and transcription and in part this may be occurring through the introduction of histone methylation marks previously associated with repressive cellular chromatin [56]. Finally, the CCCTC-binding protein (CTCF) has been shown to affect transcription from the HPV early region during lytic infection by binding to a site in the E2 open reading frame [57]. Although CTCF is not generally considered one of the typical epigenetic regulatory factors like histone modifications, it appears to play a role in regulating transcription by directly interacting with chromatin in a number of viruses including HPV [58].

## 6. Hepatitis B

Hepatitis B virus (HBV), a significant human pathogen, exists as a 3.2 kb single strand of DNA in the virus particle and as a covalently closed circular DNA (cccDNA) in the infected nucleus of a cell [15]. Not surprisingly, the cccDNA associates with histones and other cellular proteins to form a minichromosome in the infected cell [59]. Because there was a recent extensive review [60] of HBV epigenetics, this section will only focus on two aspects that are likely to be the basis for many future studies.

Using a modified ChIP-Seq procedure with enhanced sensitivity, the location of nucleosomes containing specific histone modifications and RNA PII were mapped to the HBV genome in cccDNA chromatin [61]. These studies showed that the HBV minichromosome contained nucleosomes and histone modifications similar to those seen in cellular chromatin such as H3K4me3 located near enhancer regions and acetylated histones in transcribed regions. Moreover, there were certain differences observed when comparing the organization of HBV chromatin from different infected sources, suggesting that the chromatin structure of the minichromosome was reflecting the epigenetic milieu of the infected cell to some extent. Because HBV DNA becomes chromatin upon infection the virus does not appear to be capable of transgenerational epigenetic memory. For this virus, epigenetic regulation appears to be required to control the relative proportion of mRNAs corresponding to the entire genome for its replication and the viral proteins needed for other aspects of its life cycle. Because of its significance as a human pathogen, these studies are likely to serve as the basis for subsequent investigations directed further characterization of HBV epigenetic regulation with the intention of identifying targets for therapeutic intervention.

Similarly, it seems very likely that there will be further studies on the role of the HBV protein HBx which appears to function as an epigenetic regulator to dysregulate a number of cellular genes as well as regulate the virus (reviewed in [62]). HBx appears to dysregulate a number of cellular pathways in part by binding to genomic DNA [63], changing expression patterns of miRNAs [64], affecting histone methyltransferases [65], binding to SIRT1 to activate transcription [66], and cooperating with histone methylases and demethylases to change the cell expression pattern [67].

## 7. Herpesviridae

Herpesviridae are a family of relatively large double-strand DNA viruses approximately 160 kb in size [15]. Because a number of the members of this family are significant human pathogens—including herpes simplex virus (HSV), Epstein–Barr virus (EBV), and Kaposi’s sarcoma-associated herpesvirus (KHSV)—the regulation of the life cycles of these viruses has been extensively studied. As a result, there have been a number of recent reviews focusing on epigenetic regulation in these viruses. The reader is encouraged to consult these reviews for a detailed description of regulation including dysregulation of host cells by a particular virus.

There are two aspects of the life cycle of the typical herpesvirus which are particularly relevant to epigenetics [68]. The first is the circularization and chromatinization of the linear viral DNA upon infection and entry into the nucleus of a cell. The second is the regulation of the choice between a latent and a lytic infection following infection by these viruses. The formation of chromatin structure on infecting viral DNA is a critical first step in ensuring that only relevant early gene expression occurs during the initial stages of infection. Since viral DNA contains a large number of genes with a corresponding number of promoters, without the formation of chromatin it is unlikely that only the early viral genes would be expressed.

Depending upon the cellular environment, infection by herpesviruses can result in either a lytic infection, in which, the infected cell is ultimately killed, or a latent infection in which the virus exists as an episome (minichromosome) stably in the nucleus of the infected cell. To exist as a stable episome, the virus must be able to coordinate its own replication with that of the cell and to segregate itself along with the chromosomes of the cell following replication. This aspect of regulation is generally controlled by a viral protein, which interacts with the cellular chromosomes to tether the newly replicated virus for segregation along with the chromosome. Each of the viruses has its own multifunctional protein to accomplish this. Finally, there must be a way for the virus present in a latent infection to reactivate to generate a lytic infection usually as a result of a change in the cellular environment. Epigenetics is thought to contribute to all of these processes.

KSHV has been extensively studied because of its ability to cause cancer in AIDS patients. There are recent reviews that the reader is encouraged to consult for details on the role of epigenetic regulation in this virus [69,70,71]. Epigenetic regulation of this herpes virus can be considered a model for all of the viruses. Upon infection, the linear DNA is circularized and chromatinized relatively quickly. The chromatin structure initially is organized to allow for immediate–early transcription [70] with corresponding histone modifications activating the appropriate genes and deactivating other genes. The critical RTA transcription factor which is involved in activating the lytic form of the virus is repressed shortly after infection but placed into a bivalent chromatin state containing both activating and repressive histone modifications. Latency Associated Nuclear Antigen (LANA), the latency regulator [71] is produced along with a small number of other early gene products and together are responsible for maintaining the latent state through replication and episome segregation along with cellular chromosomes. LANA functions in part by binding to the viral DNA and also to cellular chromatin [71]. Upon reactivation of the viral episome, there is a gross change in chromatin structure consistent with the activation of the late viral genes necessary for the completion of the virus life cycle and the synthesis of new virus particles [70]. In KSHV, there is also evidence that over time gene silencing in repressive chromatin may also occur through DNA methylation and interactions with noncoding RNAs [69].

Both HSV and EBV seem to be epigenetically regulated similarly to KSHV although the viral proteins responsible for the regulation are unique to the other viruses. In lytic infections, HSV viral DNA is chromatinized within 1–2 h of infection and the chromatin is acted upon by transcription factors from the virus and cell along with chromatin remodeling factors to initiate immediate–early gene expression (reviewed in [68,72]). The chromatin modifications include changes in histone modification and nucleosome positioning. In contrast in cell, which support a latent infection, chromatization appears to occur much more slowly and the genes responsible for completion of a lytic infectious cycle appear to exist as heterochromatin with typical histone modifications characteristic of heterochromatin [68,72]. The epigenetic regulation of EBV has been of particular interest because of EBV’s role in causing certain human cancers. Because of this, it has undergone some very extensive analyses including a characterization of the location of all of the typical histone tail modifications, the binding sites for a number of common transcription factors, and RNAPII on the EBV genome [73]. The results of these analyses were generally consistent with the reported associations between certain histone modifications and gene expression or repression. For example, methylated H3K4 was located at sites of gene expression and the location of RNAPII. However, the results were also somewhat confusing since repressive modifications were also associated with some of these sites [73]. The results suggested that there might be more heterogeneity and dynamic modification taking place in the viral chromatin. An overview of epigenetic regulation of EBV, including DNA methylation and other cellular processes, has also appeared recently [74]. In general, DNA methylation of the EBV genome, like its cellular counterpart, occurs to silence genes. However, there are significant differences within various latency types with respect to the genes silenced [74]. A role for CTCF in the epigenetic regulation of EBV has also been established. CTCF appears to prevent the spread of repressive histone modifications into genomic regions, which would normally be activated for transcription [74]. Finally, the review discusses the epigenetic dysregulation of the host cell genome as might be expected for a virus infection.

## 8. Future Research Directions

Our understanding of epigenetic regulation is poised for significant advancement in the next few years. One way, this will be occurring is by moving away from interpretation by association to a more mechanistic view of regulation. As described above, much of what we know about epigenetic regulation is based upon studies, which can only show that a particular epigenetic regulatory change is associated with an activating or repressive biological event. For example, a comparison between the chromatin structure of a silent gene and its active counterpart shows the changes associated with activation. This has led to some of the general regulatory rules described above, such as the observation that methylated H3K4 is associated with gene activation. Recent studies with ChIP-Seq have shown that at best this is an overly simplistic view of the role of histone modification. What is lacking in many cases is exactly how and why a particular modified histone or similar epigenetic change contributes to a biological event like the activation or repression of a specific gene. This is made more evident in the studies described in which the locations of various histone modifications are mapped to nucleosomes in chromatin. While the technology to address epigenetic mechanisms is probably available at this time, it is likely that new strategies will need to be developed to study mechanisms. This may entail new model systems, which can be exploited in vitro.

A second area where progress is likely to occur is in better understanding the relationship between the location of nucleosomes containing specific histone modifications and the binding of regulatory factors. As indicated above, some of the results to date are paradoxical. For example, in SV40, T-antigen binding to Site I results in the introduction of a nucleosome which is located essentially in the same position as Site I. It is difficult to imagine how both T-antigen and the nucleosome can occupy the same location in the same chromatin given our knowledge of how each binds to DNA. Similarly, in Hepatitis B Virus, RNAPII binds to its promoter at a site, which is overlapped by a nucleosome containing H3K4me1. Again, it is not clear how the RNAPII can bind to its target DNA if the site is also being occupied by a nucleosome.

Studies on the epigenetic regulation of DNA viruses will continue to be of significant interest for a number of reasons. First, their dependence on host cell proteins and processes for their molecular biology means that they can serve as useful models for cellular epigenetic events. More importantly, because of their general small size and ease of genetic and other forms of manipulation, they may serve as the chromatin substrates for the in vivo and in vitro studies needed to unravel the mechanisms involved in epigenetic regulation. Importantly the relatively small size of these viruses and the relatively large amounts of chromatin that can be obtained by infection, makes ChIP-Seq and similar NGS analyses cost effective. In addition, the high resolution, which can be obtained from the analysis of these viruses, will also be important for the mechanistic studies. As indicated above with SV40 we can obtain single base resolution in our ChIP-Seq studies. With this high resolution, it is possible to further characterize the small shifts in nucleosome location, which our group and others have recently observed.

A better understanding of the mechanisms responsible for the introduction of epigenetic information and, correspondingly, the mechanisms responsible for reading this information may possibly lead to novel treatments for infections by these viruses many of which are serious human pathogens. Characterizing the mechanisms that underlie each form of epigenetic regulation and dissecting the complex interplay between viral DNA sequences may identify viral proteins and cellular contributors that will lead to new targets for therapeutic intervention.

## Figures and Tables

**Table 1:** Functional outcomes of epigenetic regulation.

Epigenetic Process	Target	Function
DNA Methylation	DNA in cell or viral epigenome	Typically silence genes
Nucleosome Location	Cellular and Viral Chromatin	Nucleosome position controls access to DNA sequences
Histone Modification	Cellular and Viral Chromatin	Histone acetylation and methylation on H3K4/H3K36 association with activation of transcription, methylation on H3K9 and H3K27 is associated with repression, methylation of H4K20 could result in either activation or repression
Histone Variants	Cellular and Viral Chromatin	Not yet well understood for viruses
miRNA	Cellular and Viral Chromatin	Modify gene expression at the level of translation
